# Identification of CT-based non-invasive Radiographic Biomarkers for Overall Survival Stratification in Oral Cavity Squamous Cell Carcinoma

**DOI:** 10.21203/rs.3.rs-3263887/v1

**Published:** 2023-08-23

**Authors:** Xiao Ling, Gregory S. Alexander, Jason Molitoris, Jinhyuk Choi, Lisa Schumaker, Ranee Mehra, Daria A. Gaykalova, Lei Ren

**Affiliations:** 1 Department of Radiation Oncology, University of Maryland School of Medicine, Baltimore, Maryland, USA; 2 Department of Breast Surgery, Kosin University Gospel Hospital, Busan, KR; 3 Marlene and Stewart Greenebaum Comprehensive Cancer Center, University of Maryland School of Medicine, Baltimore, MD, USA; 4 Institute for Genome Sciences, University of Maryland School of Medicine, Baltimore, Maryland, USA; 5 Department of Otorhinolaryngology-Head and Neck Surgery, Marlene & Stewart Greenebaum Comprehensive Cancer Center, University of Maryland Medical Center, Baltimore, Maryland, USA; 6 Department of Oncology, Sidney Kimmel Comprehensive Cancer Center, Johns Hopkins University, Baltimore, Maryland, USA

## Abstract

This study addresses the limited non-invasive tools for Oral Cavity Squamous Cell Carcinoma OSCC survival prediction by identifying Computed Tomography (CT)-based biomarkers for improved prognosis. A retrospective analysis was conducted on data from 149 OSCC patients, including radiomics and clinical. An ensemble approach involving correlation analysis, score screening, and the Sparse-L1 algorithm was used to select functional features, which were then used to build Cox Proportional Hazards models (CPH). Our CPH achieved a 0.70 concordance index in testing. The model identified two CT-based radiomics features, Gradient-Neighboring-Gray-Tone-Difference-Matrix-Strength (GNS) and normalized-Wavelet-LLL-Gray-Level-Dependence-Matrix-Large-Dependence-High-Gray-Level-Emphasis (HLE), as well as smoking and alcohol usage, as survival biomarkers. The GNS group with values above 14 showed a hazard ratio of 0.12 and a 3-year survival rate of about 90%. Conversely, the GNS group with values less than or equal to 14 had a 49% survival rate. For normalized HLE, the high-end group (HLE > −0.415) had a hazard ratio of 2.41, resulting in a 3-year survival rate of 70%, while the low-end group (HLE <= −0.415) had a 36% survival rate. These findings contribute to our knowledge of how radiomics can be used to anticipate the outcome and tailor treatment plans from people with OSCC.

## INTRODUCTION

Oral Cavity Squamous Cell Carcinoma (OSCC) is an aggressive site for malignancies of the head and neck Squamous Cell Carcinoma (HNSCC) with a poor prognosis. Despite improvements in surgical techniques and adjuvant therapies the 5-year overall survival rate hovers between 30-50%, depending on the stage and recurrence status of the disease ^1^. It also has a substantial impact on public health worldwide ^2–4^, with millions of new cases reported annually. For patients with resectable disease, surgery is the standard of care with adjuvant treatments recommended depending on pathologic features and individualized risk of recurrence. In patients more advanced stages or adverse pathologic features radiotherapy with or without the addition of cisplatin-based chemotherapies are often recommended. Adjuvant radiotherapy comes with significant detriments to quality of life which are exacerbated by the use of concurrent chemotherapy. It is, therefore, crucial to identify patients who are at a higher risk of local recurrence to increase the therapeutic window with the use of prognostic and predictive biomarkers. Additionally, it may help to identify patients who are at higher risk of distant metastatic spread who may benefit from novel systemic agents that may reduce the risk of spread. By utilizing these biomarkers, patients’ cancer trajectory can be better estimated, enabling medical professionals to tailor treatment plans accordingly. Early access to personalized therapies for high-risk patients can positively impact their prognosis and improve their overall outcomes.

Histological biopsies are commonly used for OSCC diagnosis and prognosis evaluation ^5^. Plus, biological fluids-based measurements collected from saliva, blood, serum, and plasma have also been explored as diagnostic and prognostic biomarkers for OSCC. Numerous studies ^6–8^ have validated the dysregulation of specific miRNAs in OSCC, including miRNA-184 ^6^, miR-31 ^9^, and miR-27b ^10^, which are associated with malignant transformation and disease progression. Additionally, elevated levels of biomarkers such as CA125 ^11,12^, tissue polypeptide antigen, and Cyfra 21-1 have been observed in OSCC patients compared to control groups. Molecular biomarkers including p16 ^13^, EGFR ^14^, TP53 ^15^, and Cyclin D1 ^16^ have also demonstrated their ability to distinguish between patients and control groups and show disparities in overall survival based on cutoff values.

While these biomarkers evaluated in pre-clinical settings hold promise in enhancing disease detection, prognosis, and personalized treatment, several challenges persist. The technical complexity involved in extracting and analyzing biomarkers, such as H&E staining, the tissue microarray, and sequencing, often results in high costs and the need for specialized resources. As a result, their applicability could be limited in certain contexts. Issues related to reproducibility and standardization across different laboratories, as well as risks of false positives and negatives, further complicate their utility. Additionally, the validation process of a biomolecule-based assay, from discovery to clinical application, is typically laborious and time-consuming, with many potential markers failing to demonstrate efficacy in diverse populations ^17^. Even more, minimizing the total package time are essential to prevent adverse effects on patients in need of adjuvant therapy, allowing for timely initiation of appropriate treatments. Furthermore, some biomarkers, despite indicating the presence of a disease, may not provide actionable insights for treatment strategies, thus limiting their clinical utility ^18,19^. Finally, the use of genetic and other biomolecule biomarkers raises ethical, legal, and social considerations ^20^. That explains why thos biomarkers did not reach the clinical setting yet and were not FDA-approved.

The integration of imaging modalities-based biomarkers, such as Computed Tomography (CT), Positron Emission Tomography (PET), and Magnetic Resonance Imaging (MRI), enables the extraction of quantitative imaging features known as radiomics features. In contrast to biomolecule-based assays, imaging techniques are non-invasive. The imaging information is readily available from routine diagnostic scans without incurring additional costs. Moreover, imaging provides unique 3D information about the tumor. These radiomics features can be leveraged to develop predictive models for survival and treatment failure. The rationale behind this approach is that these images capture crucial information about the tumor phenotype and microenvironment ^21^. Recent studies have highlighted the potential of medical images in defining genetic mutations related to cancer and utilizing radiomics-based imaging biomarkers for outcome predictions ^22–24^ Outcome prediction typically involves the use of classical statistical methodologies or Artificial Intelligence (AI) models trained on historical data encompassing genomic, epigenomic, and other omics data. These models aim to uncover patterns or predictors associated with the specific outcome of interest, enabling personalized predictions for individual patients.

Over recent decades, Imaging biomarkers (IBs) have demonstrated their capacity to furnish accurate prognostic information for treatment outcomes across a variety of diseases trajectories ^25,26^, including cancer ^27^. The pervasive use of CT, PET, and MRI biomarkers in cancer research can be attributed to their non-invasive nature and their ability to portray the multifaceted nature of heterogeneous tumors ^28^. Studies have shown the clinical utility of CT and PET in predicting treatment outcomes for rectal and lung cancer patients ^24,29^. Recent investigations also hint at the potential role of PET imaging in identifying cancer-associated genetic mutations ^30^ and the prospective use of radiomics-based imaging biomarkers for outcome predictions in head and neck cancer ^31^. However, the identification of reliable imaging biomarkers for overall survival (OS) prediction in OSCC patients remains lacking. The difference between our study and previous research is elucidated in Feature Selection section (Materials and Methods). This study aims to identify CT imaging biomarkers for OS prediction in OSCC patients by employing a novel machine-learning (ML) framework.

A single institution academic health network, serving a diverse population, possesses a diverse cohort of oral cavity squamous cell carcinoma cases. This study endeavors to identify imaging biomarkers associated with overall survival in oral cavity squamous cell carcinoma patients. We employed a retrospective study design using high-dimensional radiomics data and clinical follow-up information. The primary endpoint of the study was overall survival. We used the Cox Proportional Hazard model (CPH) to achieve the goal. Given the high dimensionality of the imaging data, the feature selection methods, in conjunction with Best Subset Selection (BSS) strategy, were deployed to avoid overfitting and to select a parsimonious set of candidate factors. In addition, we also investigated the variations in overall survival outcomes by stratifying individuals based on different levels of two identified radiomics biomarkers, respectively. Lastly, the final CPH model was summarized in a nomogram to facilitate the treatment decision.

## Results

### Feature Selection

In this study, we employed three independent feature selection methods to create a refined input dataset for Cox proportional hazard modeling, addressing various issues posed by high-dimensional data. By pruning features based on the Pearson correlation coefficient and Cramer’s V score, we reduced the initial 1,092 radiomics features to 79 and the 7 categorical features to 6. Subsequently, a score screening procedure further narrowed down the radiomics features to 17. Finally, the SparseL1 algorithm was fine-tuned to select seven features, resulting in an active input dataset size of 13. The feature selection process effectively mitigated multicollinearity, reduandancy, and computational complexity. The final 10-fold concordance index demonstrated the efficacy of this approach.

### Best Subset selected CPH model

In the 10-fold cross-validation of the final Cox model, the results in Table 1 consistently demonstrated a strong model fit, as evidenced by the log-likelihood ratio and score test.

Both tests yielded significant p-values across all folds, with average p-values of 3e-06 and 6e-06 for the log-likelihood ratio and the score test, respectively, suggesting that our model is highly significant and provides a good fit to the data. The training concordance index (CI)remained stable and high across all iterations, with an average value of 0.727 (SD = 0.011). The testing CI has a mean value of 0.699 and a standard deviation of 0.103.

This CI, an indicator of predictive accuracy, demonstrates the effectiveness of this prognostic model. The p-values for the continuous variables Gradient-NGTDM-Strength (GNS), Wavelet-LLL-GLDM-LargeDependenceHighGrayLevelEmphasis (HLE), and Stage demonstrated varying levels of significance across the cross-validation folds. GNS (mean p-value = 0.015, SD = 0.013), HLE (mean p-value = 0.023, SD = 0.012), and Stage (mean p-value = 0.019, SD = 0.016) all showed significance in the model to varying degrees. The covariate ETOH represents alcohol usage status, with three categories: “Alcohol user” (category 1, reference level), “Alcohol non-user” (category 2), and “Unknown” (category 3). The average p-value for the 10-fold cross-validation of the non-user categroy is 0.084, with a standard deviation of 0.057 This suggests that alcohol usage may have a significant influence on the model, although the effect might not be as robust as the continuous variables, given there are 18 missing values in ETOH. Table 2 shows the final CPH model parameter estimates and goodness of fit statistics. For instance, the hazard ratios in column *H.ratio* provide insight into the risk effect of each factor. For the clinical ETOH effects, a hazard ratio of 0.54 indicates that nonusers have a hazard of 0.54 times that of users. In terms of odds, the probability of death occurring (P) can be calculated by P = H.ratio / (1 + H.ratio). For alcohol users, there is a 65% chance of death, while for non-users, there is a 35% chance of death. The 95% confidence interval (CI) for the effect of non-users lies between 0.29 and 1.01, indicating an acceptable variability in the hazard ratio of 0.54. We also investigated the association between stage, treated as a ordinal variable, and survival time. The hazard ratio for stages is 1.37, indicating a 37% increase in risk when moving from one stage to the next. By standardizing HLE to HLE_s_ (zero mean with a standard deviation of 1), the Hazard ratio of 1.29 for HLE_s_ indicates that the chance of death increases by 1.29 times for patients with one standard deviation higher HLE_s_ compared to the previous HLE_s_. In other words, for each unit increase in HLE_s_, there is a 14% increase in the chance of death compared to the previous one. The lower bound of 95% confidence interval [1.05, 1.58] is also greater than 1, further suggesting that HLE_s_ is a significant risk factor for overall survival in OSCC. On the contrary, GNS has both a hazard ratio of less than 1 and a 95% confidence interval. The hazard ratio of 0.94 suggests a 52% chance of death for a patient with one unit increase in GNS, compared to a 48% chance of death for a patient with no increase in GNS. Interestingly, even when we include the stage as a covariate in the multivariate analysis, we still identify these two significant radiomics features. This suggests that there are distinct survival differences associated with these features, as demonstrated by the Kaplan-Meier estimator.

### Stratification Analysis

**Figure 1.**
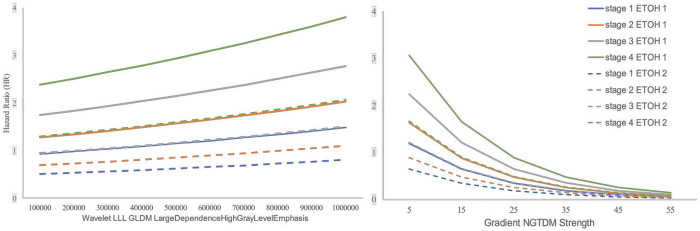
Hazard Ratio functions of radiomics features at diagnosis on each state transition, stratified by ETOH (solid versus dashed lines) and cancer stage (various colors).

The Hazard Ratio in Figure 1 illustrates the impact of two radiomics features HLE and GNS, on each stage, stratified by ETOH and cancer stage while keeping other covariates fixed at their sample means. The KM curves are stratified based on drinking status (drinker and non-drinker) across different cancer stages. Each curve represents the estimated hazard ratio for individuals in a specific group (e.g., drinkers with stage 1 OSCC, and non-drinkers with stage 2 OSCC). The vertical axis represents the hazard ratio, while the horizontal axes represent the radiomics features values. The left plot demonstrates that a higher Hazard Ratio is linked to advanced stage status, with a positive correlation between HLE and Hazard Ratio when other factors are held constant. Alcohol users (ETOH=1) exhibit a consistently higher hazard ratio across all stages than non-users (ETOH=2). In the right plot, we observe a negative relationship between GNS and Hazard Ratio. The hazard ratio shows a steadier change for non-users at each.

Several trends are notable in this comprehensive dataset reflecting the clinical characteristics of two stratified cohorts, respectively, designated as HLE_l_ vs HLE_h_ and GNS_l_ vs GNS_h_. The low-end group (GNS_l_) consists of patients with a GNS value less than or equal to 14, while the high-end group (GNS_h_) comprises patients with GNS values greater than 14. Similarly, for the normalized HLE (HLE_s_) feature, we formed two groups: the low-end group (HLE_l_) with an HLE value less than or equal to −0.415 and the high-end group (HLE_h_) with HLE_s_ values greater than −0.415. This stratification allows us to identify differences in outcomes and treatment response between the groups, as well as explore the potential predictive value of these radiomics features. Predominantly male participation is observed in both cohorts, with the low-end cohort significantly outweighing the high-end cohort. The mean age at diagnosis is almost identical in both cohorts. As for lifestyle habits, low-end cohorts have a slightly higher percentage of smokers. Regarding alcohol consumption, a larger 73% of GNS_h_ participants were alcohol users compared to 45% in GNS_l_. The alcohol consumption rate was distributed evenly in HLE groups.

**Figure 2.**
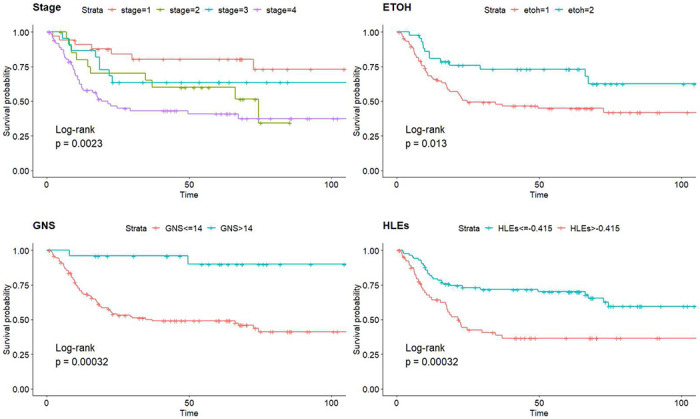
The top two KM curves represent overall survival rates stratified by each baseline factor, while the bottom two KM curves depict the survival rates stratified for two radiomics features: GNS and scaled HLE.

Figure 2 displays the Kaplan-Meier curves for each feature. Stratification by GNS reveals a significant difference in overall survival between the two groups. The group with GNS greater than 14 shows a flat survival curve with only two events out of 24 occurring, suggesting that this group generally has a good prognosis with a high survival probability over the follow-up period. In contrast, the median overall survival for the group with GNS less than 14 is approximately 37 months, with 64 events out of 125. The 3-year survival rate for the group with GNS greater than 14 is around 90%, compared to 49% in the group with GNS less than 14. For HLE, the 149 patients were divided into two groups: those with HLEs less than 1,370,000 and those with HLEs greater than 1,370,000. The median overall survival for the group with HLEs less than 1,370,000 is 154 months, compared to 10 months for the group with HLEs greater than 1,370,000. The 3-year survival rate is 60% for the former group and 23% for the latter.

#### Radiomics-Based Nomogram

**Figure 3.**
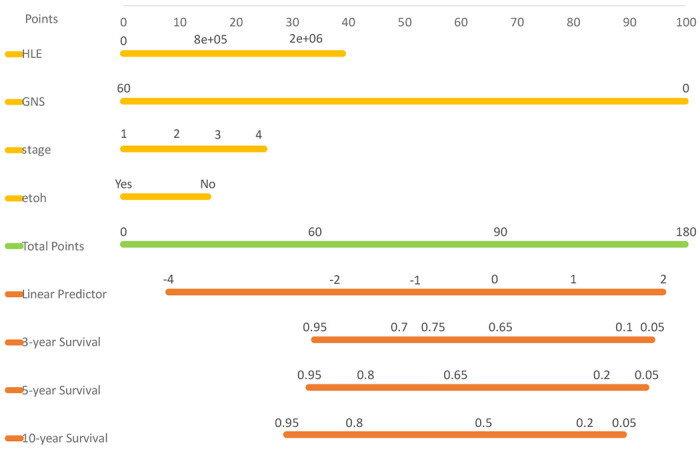
Nomogram from the fitted Cox model.

Based on our model, we developed a nomogram in Figure 3 that visually represents the CPH model presented in Table 2. This nomogram allows the estimation of overall survival for OSCC patients after treatment. To use the nomogram, one simply needs to input the values of four variables (HLE, GNS, stage, and ETOH) and mark them on their respective axes. Connecting these marked values with vertical lines to the top scale (points scale) determines the points for each variable. Adding these points together and marking them on the total points axis provides the total points. By connecting the position of total points with the corresponding survival probability, one can estimate the overall outcomes based on the Linear Predictor.

The purpose of the nomogram in thIs study is to provide a practical and user-friendly tool for estimating overall survival in OSCC patients after treatment. By integrating multiple prognostic factors into a graphical representation, the nomogram allows healthcare professionals to easily assess individual patient outcomes and make informed decisions regarding treatment strategies. The benefit of using a nomogram lies in its ability to incorporate complex statistical models into a visually intuitive format, enabling personalized risk prediction. It offers improved prognostic accuracy, individualized treatment planning, and enhanced communication between healthcare providers and patients. The nomogram serves as a valuable addition to clinical practice by facilitating shared decision-making and promoting precision medicine approaches in the management of OSCC.

## Discussion

The present study aimed to evaluate the prognostic value of radiomics features in oral cavity squamous cell carcinoma (OSCC) patients. Our findings demonstrate that radiomics analysis of pre-treatment CT scans can provide valuable insights into the factors influencing survival and serve as prognostic biomarkers in this patient population.

Key findings of our study include two significant hazard ratios, 1.29 and 0.94, between two radiomics features and overall survival. The concordance-index (CI) showed a stable and high average value of 0.7, indicating good predictive accuracy of the prognostic model. These results highlight the potential of medical imaging, particularly radiomics, as a non-invasive and quantitative method for treatment prognosis. Methodological considerations were also addressed in our study. We standardized the voxel spacing across patients by resampling CT images to ensure accurate feature calculation. Additionally, gray-level normalization was applied to enhance the comparability of features and improve their robustness against variations in different settings. These steps are crucial for reliable and reproducible radiomics analysis. The feature selection process in our study involved sequential approaches consisting of Correlation Analysis, Score screening, and the SparseL1 algorithm. This process effectively reduced the dimensionality of the feature space while retaining a significant amount of the prognostic information present in the original data. This approach helps to mitigate issues such as bias, overfitting, and multicollinearity that can arise in high-dimensional data analysis. Best subset selection modeling techniques were utilized to identify optimal Cox proportional hazards models, leading to the identification of biomarkers associated with survival in OSCC patients.

The Cox proportional hazards modeling revealed several significant radiomics features associated with survival in OSCC patients. The continuous variables, Gradient-NGTDM-Strength (GNS) and Wavelet-LLL-GLDM-LargeDependenceHighGrayLevelEmphasis (HLE), showed varying levels of significance in the model, indicating their potential as prognostic biomarkers. The categorical variable, alcohol consumption (ETOH=2), also demonstrated some degree of influence on the model. The performance of our Cox model was assessed using log-likelihood ratio and score tests, which consistently yielded small p-values across all folds in the 10-fold cross-validation. The concordance index (CI), a measure of predictive accuracy, remained stable and high, indicating the effectiveness of our model. These results suggest that our Cox model provides robust predictive performance for survival in OSCC patients.

While our study highlights the potential of radiomics in OSCC prognostication, it is important to acknowledge its limitations. The relatively small sample size and the nature of the survival study may impact the stability of the validated radiomics features in our model. To support a low-biased and variance survival model with four effects, it is recommended to have at least 40 events in each training set, requiring a sample size containing 67 events if 60% is allocated for the training set. This criterion restricts the degrees of freedom our model can reach, potentially affecting the prognostic ability of the underlying radiomics. Additionally, the current analysis focused on extracting radiomics features from a single imaging modality, i.e. CT. Future studies are warranted to investigate radiomics features from multiple modalities, such as CT and MRI, which opens up the potential to improve the prediction accuracy further. Last, the significant association between certain radiomics features and overall survival suggests that imaging features may reflect some of the underlying molecular characteristics of the tumors. Future investigations are warranted to integrate genetic TP53 mutations ^15^ and P16 overexpression ^32^ and radiomics data to characterize squamous cell carcinoma of the head and neck and provide an alternative non-invasive, multi-modal approach to OSCC outcome predictions.

In conclusion, our study demonstrated the potential of radiomics as an effective tool to predict treatment response in OSCC patients. Incorporating radiomics analysis into clinical practice could improve decision support and enhance patient stratification, reducing both over-treatment and under-treatment to improve outcomes. The findings from the study pave the way for future investigations through a larger clinical trial to further evaluate the clinical efficacy of radiomics biomarkers for overall survival prediction for OSCC patients.

## METHODS

### Endpoints of Interest and Study Cohorts

This retrospective cohort study examines a group of oral cavity squamous cell carcinoma (OSCC) patients who underwent contrast-enhanced CT scans at the institution between 2006 and 2017. The sample size consisted of 149 patients. We collected six clinical attributes, including age at diagnosis, gender, tobacco use, alcohol consumption, stage, and race, summarized in Table 3. Table 1 presents a comprehensive summary of the clinical factors observed in this cohort. The mean age at the diagnosis was 62, ranging from 29 to 98 years’ old. Patients were categorized into four stages (I, II, III, and IV ) based on the pathological assays of tumor specimens. Smoking and alcohol status were self-reported and coded as 1 for yes and 2 for no. The missing values for smoking and alcohol status were hard-coded as 3 due to their substantial representation within the dataset. Six treatment modalities, including chemoradiotherapy (CRT), chemotherapy (CT), surgery (Sx), radiotherapy plus surgery (RT+Sx), CRT+Sx, and CT+Sx, were administered to patients as their initial treatment. The endpoint in this study was overall survival (OS), defined as the time from the date of diagnosis (determined by the diagnostic scan or biopsy) to the date of death or last follow-up day. As of November 04, 2019, a total of 66 patients died after treatment. The average survival time among all 149 patients was 40 months (ranging from 1 to 154 months). Among the patients who died, the average survival time was 19 months (ranging from 2 to 154 months), whereas among patients alive at the last follow-up the average survival time was 58 months (ranging from 1 to 137 months).

### Data preparation and overall workflow

**Figure 4.**
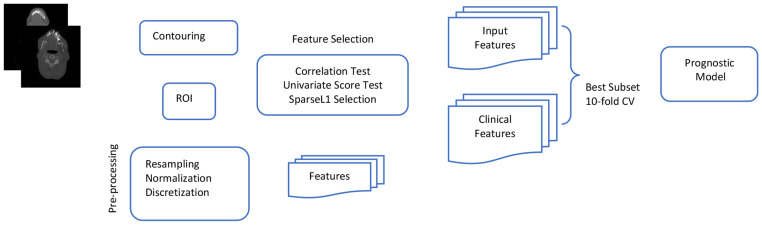
Image feature extraction and outcome prediction workflow.

**Figure 5.**
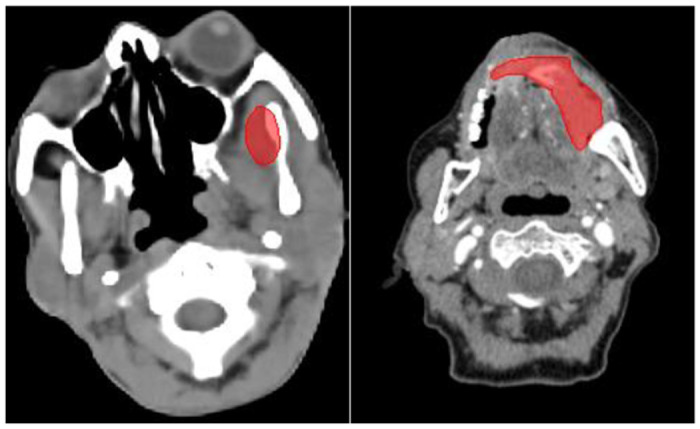
ROI (red) in the left oral cavity.

This study aims to enhance the prognostic and predictive value of radiomics for OSCC patients by extracting1,092 radiomics features from pre-treatment CT scans. The primary objective is to identify prognostic and predictive biomarkers within these CT scans that can facilitate the assessment of treatment effectiveness at the individual patient level. This will enable the selection of tailored treatment strategies and ultimately lead to improved patient outcomes. The workflow outlining our approach is illustrated in Figure 4. In this workflow, the tumor volume serves as the region of interest (ROI) from which all radiomics features are computed (as shown in Figure 5). The contouring of the ROI was performed manually by experienced Radiation Oncologists using the Varian Medical System Eclipse software environment. These features underwent a selection process to minimize redundancy and were combined with clinical data. A CPH model, optimized via the best subset approach and 10-fold cross-validation, was then applied. The model’s predictive performance was evaluated using the concordance index. All statistical analyses were performed using R programming language, with a significance level (alpha) set at 0.05 for all tests. All procedures included in this application have been approved previously by the institutional review boards (IRB) of the University of Maryland School of Medicine Institutional Review Board.

### Pre-processing

It is worth noting that a previous study ^33^ highlights that radiomics features are sensitive to voxel size. Therefore, maintaining consistent voxel sizes across patients is crucial to obtain accurate and reliable radiomics feature calculations. In this study, CT resolution sizes varied from 0.3×0.3×0.5 to 1.3×1.3×5. We resampled all CT images to the resolution of 1x1x1mm^3^ using the basis spline algorithm (Bspline) to interpolate the HU values in the resampled voxels. Correspondingly, we used the nearest neighbor algorithm to resample the tumor contours to the same resolution. The subsequent procedure, gray level normalization, is critical in improving the comparability and robustness of radiomics features across different settings and variations among patients. As demonstrated in a previous study ^33^, gray level normalization reduces the variance and enhances the robustness of radiomics features, particularly regarding varying discretization levels. Therefore, normalization was applied to scale the Hounsfield Unit (HU) values to a uniform range across patients. This was achieved by subtracting the mean and dividing the voxel values by the standard deviation. Conventionally, this process yields an ROI with intensities approximately in the range [−3, 3] after removing outliers outside three standard deviations. These resulting values are then further scaled using a normalized scale parameter, such as a value of 100, resulting in an approximate range of [−300, 300]. Finally, the intensities within the ROI were discretized using a unified bin-width of 5, starting from the minimum normalized HU value of 0. In this study, we selected a bin width of 5 to ensure an adequate number of bins (between 1 and 400) for capturing more granular textural information ^34^. This discretization step assigns a new value to each voxel using the formula $\text{floor}(\frac{origin\;intensity}{5})+1$. This discretization approach offers the advantage of noise suppression and improved robustness of radiomics features.

### Feature Extraction

**Figure 6.**
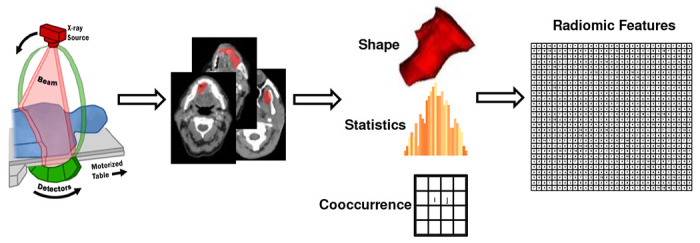
The typical workflow of radiomics feature extraction.

Features extracted from medical images carry the phenotypic characteristics of a tumor, as shown in Figure 6. A typical medical image features data set involves measurements of tens of thousands of voxel intensities for a single tumor sample; usually, the number of features (*p*) is ≫ sample size (*n*). In this study, feature extraction was performed in each set of images using the Python library PyRadiomics ^35^. Imaging Biomarker Standardization Initiative (IBSI) ^36^ described features were extracted in six families, including shape-based, first-order statistics, gray level co-occurrence matrix (GLCM), gray level run length matrix (GLRLM) ^37^, grey level size zone matrix (GLSZM) ^38,39^, gray level dependence matrix (GLDM) ^40^ and neighborhood grey tone difference matrix (NGTDM) ^41^. Additionally, features are calculated from wavelet, Laplacian of Gaussian (Log), square, square root, logarithm, exponential, and gradient-filtered images, making the total number of features 1092.

### Feature Selection

#### Motivation and Overview

In the field of time-to-event data analysis, the Lasso-Cox model ^42^ has gained popularity for its efficiency and simplicity in modeling high-dimensional data ^43^. This model allows for simultaneous regression and variable selection, making it a preferred choice in handling high-dimensional data. However, this algorithm often falls short of providing accurate predictive results. The regularization parameter in the Lasso-Cox model emphasizes penalization on large coefficients, leading to potential bias and an overly simplistic model that may underfit the data. To mitigate this issue and improve prediction accuracy, an alternative model called the Elastic-Net was introduced^44^. The Elastic-Net model incorporates both ℓ_1_ and ℓ_2_ penalties in ordinary least squares estimation and has been extended to handle time-to-event data ^45^. However, the increased complexity of the Elastic-Net model poses challenges in searching for optimal hyperparameters, requiring additional validation or resampling techniques. This increases the computational complexity and potentially results in solutions trapped in local optima. Another challenge in radiomics data is high multicollinearity, which occurs when there is a high correlation between two or more measurements in the data. For example, sphericity, minor axis length, max axis length and elongation are variables that exhibit strong multicollinearity in the sense that elongation is merely the inverse of spherical disproportion, and elongation is the ratio of the minor axis length to the max axis length. Multicollinearity can lead to the phenomenon where a variable is not deemed significant when correlated features are also present in the model. While regularization modeling techniques can partially mitigate multicollinearity, they may struggle when dealing with highly correlated variables.

Regression modeling often suffers from bias, overfitting, and numerically unstable estimation in cases where the number of predictors (p) is much larger than the number of samples (n). Therefore, feature selection plays a crucial role in this study. From a practical standpoint, it is desirable to build parsimonious prognostic models that are both effective and easy to use for healthcare professionals. This challenge also extends to Cox regression for time-to-event data. Studies ^46–48^ have emphasized the importance of developing parsimonious Cox models. We propose a feature selection approach to optimize the Cox Proportional Hazard model, consisting of several steps outlined in Table 4. Firstly, we pruned the highly correlated features.

Next, we selected a subset of features based on their relationship with overall survival. Finally, SparseL1 ^49^ recommended a final active subset, effectively eliminating redundant features to ensure computational feasibility for the Best Subset Selection strategy.

#### Pearson correlation analysis

**Figure 7.**
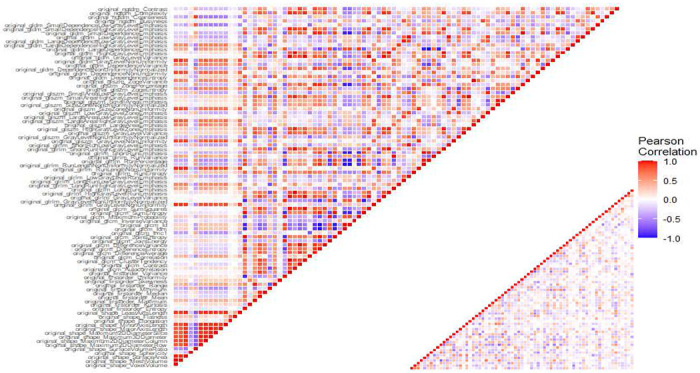
Comparison of Correlation Coefficient Heatmaps: On the left, the diagonal heatmap illustrates the pairwise correlations among features before pruning. On the right, the diagonal heatmap demonstrates the correlations after pruning. The color scale represents the strength of the correlation, with blue indicating negative correlation, red indicating positive correlation, and white representing no correlation.

**Figure 8.**
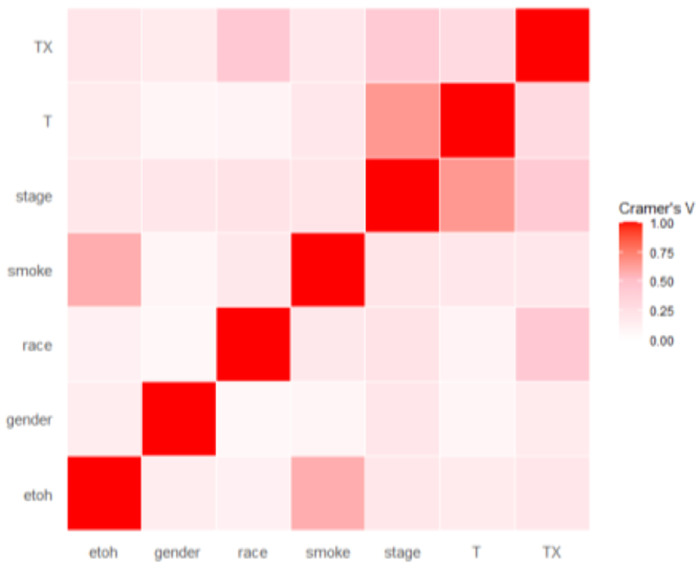
Cramér’s V for clinical features.

Given that radiomics features exhibit strong multicollinearity, relying solely on regularization modeling can introduce bias. To mitigate multicollinearity, we first employed Pearson’s correlation coefficient to detect linear dependencies among radiomics features. The Pearson correlation ranges from −1 to 1, with a value of 0 indicating no linear correlation. In the medical field, a Pearson’s score of 0.7 suggests a moderate agreement between two features based on previous studies ^50,51^. Features with a Pearson’s score exceeding 0.7 were pruned, resulting in 79 features for subsequent analysis. The result of pruning is illustrated in Figure 7. Figure 7 uses a color scheme where white represents no correlation, blue represents a perfect negative correlation, and red represents a perfect positive correlation. The left diagonal map illustrates the correlation coefficients prior to the feature selection process, revealing the initial relationships between features. The right diagonal map presents the correlation coefficients after highly correlated features have been removed, demonstrating the outcome of the feature selection process. The left diagonal map initially revealed numerous red and blue shades, indicating strong positive and negative correlations, respectively, among the data. By comparison, the right diagonal map is lighter, indicating these highly correlated features were subsequently removed from the data. To detect correlation among categorical features, we utilized Cramer’s V value. Figure 8 is a graphical representation of the Cramer’s V between categorical variables in the data, where white (Cramer’s V=0) represents no correlation and red (Cramer’s V=1) represents a perfect correlation. Feature T and Stage were found to be correlated. We dropped the T variable and retained smoke and ETOH, considering that numerous studies have identified smoking and drinking as risk factors. These correlation measures provide insights into the relationships among radiomics features and aid in addressing multicollinearity to ensure more robust and accurate modeling in our study. After pruning, 1,013 radiomics features and 1 categorical feature were effectively eliminated, resulting in a total of 85 features for subsequent analysis.

#### Univariate Score test

The univariate Cox score is the most straightforward method for identifying features associated with variability in survival time in time-to-event data analysis ^47^. Our focus is on reducing the number of radiomics features. The screening procedure consists of two steps: fitting 79 univariate Cox proportional hazards models for all radiomics features and using the score test statistic to assess the strength of association between each feature and the outcome. We prioritize p-values over setting a threshold for the statistic value. Features with a score test p-value less than or equal to 0.05 were considered significantly associated with the outcome and retained for subsequent analysis, resulting in 17 features for the next step.

#### SparseL1 Selection

To fit a CPH model, it is critical to choose an appropriate degree of freedom that balances the complexity and accuracy of the model. According to ^52,53^, a useful heuristic is to limit the number of predictors used in the fitting should be at most 15% of the events in the training sample. This criterion is corroborated by the simulations study in ^54^ that the prediction error is lower in CPH models that satisfy this condition. In our study, we observed 66 out of 149 events. Therefore, our target degree of freedom in the final model should be at most 4. Subsequently, we employed the Best Subset Selection (BSS) Modeling strategy in order to identify the best Cox Proportional Hazard (CPH) models based on their Concordance Index. BSS is widely recognized as a highly effective strategy for identifying the best parsimonious model, surpassing other strategies such as stepwise selection, forward selection, backward elimination, and Lasso. However, the computational cost associated with BSS limits its practical usage compared to other techniques. Considering fitting 2, 3, 4 degree-of-freedom CPH with an input data of 17 radiomics features plus 6 clinical features, BSS needs to estimate 41,262 coefficients at least. Exhaustively evaluating all possible subsets is computationally infeasible. Therefore, we need to reduce computational efforts by limiting the number of input variables before BSS. In this study, we employed a variation of Principal Component Analysis (PCA) known as the SparseL1 algorithm ^49^ to constrain the input data. The SparseL1 algorithm approximates the solution vector *v* by solving the NP-hard problem:

$$\underset{v,\alpha }{\min}\left|X-\alpha v\right|+\lambda \left|v\right|$$

where α^n×1^ represents the representation vector of *n* observations and *v*^1×*p*^ is a sparse vector in which each coordinate corresponds to a specific radiomics feature. By enforcing regularization, SparseL1 encourages many coordinates to be zero, effectively eliminating redundant features. SparseL1 is less sensitive to outliers compared to PCA, ensuring robust and consistent solutions. Additionally, the sparsity of the subspace can be adjusted using a single parameter λ, which serves as a controller for the number of inputs. Using this algorithm, we were able to reduce the previous 17 radiomics features to 7 features.

### Cox Modelling and Best Subset Selection

After feature selection, a multivariate Cox proportional hazards model was utilized to model the prognosis for individual patients. The Cox proportional hazards model is a commonly used approach for analyzing time-to-event data and assessing the effects of predictors on survival time. The Cox proportional hazards modeling is concerned with estimating the coefficients in the linear model:

$$\ln\frac{h\left(t|x_{i}\right)}{h_{0}\left(t\right)}=\beta x_{i}$$

where $h\left(t|x_{i}\right)$ is the cumulative hazard function for subject *i* with *m* variables, under the assumption the hazard ratio comparing any two observations remains constant over time. The coefficients β were estimated by solving maximizing the partial likelihood function:

$$ℒ(\beta )=\prod_{r\in E}\frac{\exp\;(\beta x)}{\sum_{i\in {\bar{E}}_{r}}\;\exp\;\left(\beta x^{i}\right)}$$

where *E* is the set of indices of dead patients and ${\bar{E}}_{r}$ is the set of the indices of alive patients at the time $t_{r}$. We employ the Concordance index as the primary criterion for Best Subset Selection:

$$\underset{\beta }{\max}\sum_{i=1}^{n}P\left(\beta x_{i}>\beta x_{j}|T_{i}<T_{j}\right)\;\mathrm{s.t.}|\beta {|}_{0}\leq k$$

where $|\beta {|}_{0}$ is the $\ell_{0}$-norm of β. Finally, a 10-fold cross-validation procedure was used to assess how the selected model will generalize to a new data set. The data was split into 10 folds, ensuring an even distribution of status and race within each fold. The training set model, represented by ${\hat{\beta }}_{\mathrm{train}}$ was used to predict the risk factors of the testing set, and the concordance index was calculated and averaged across all folds.

**Figure 9.**
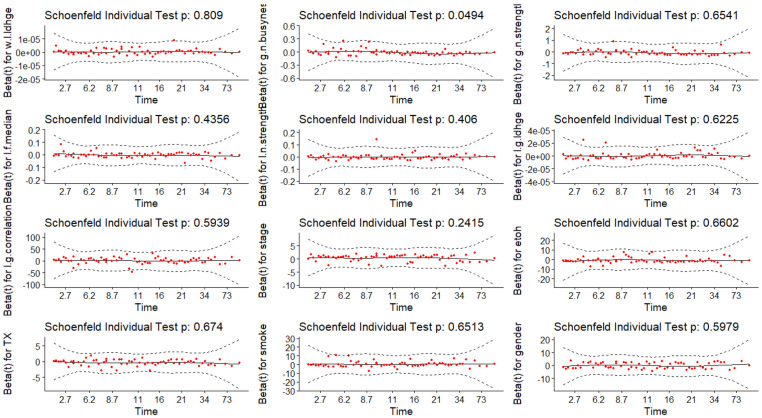
Hazard Proportional Assumption Test. Global Schoenfeld Test p-value=0.594

To validate the assumption, Schoenfeld tests were conducted, and a graphical examination was performed by observing changes in the effect over time. In Figure 9, the solid line represents a smoothing spline fit to the plot, with the dashed lines depicting the 95% confidence band around the fit. Notably, all splines remain within this band without any discernible pattern over time, indicating no changes in the effects. Additionally, Schoenfeld tests at both individual and global levels do not provide sufficient evidence to reject the proportional hazards assumption. Thus, strong statistical support is obtained for the assumption of hazard proportionality across all effects. CPH models are fitted by considering all combinations of 13 features. The Likelihood Ratio Test, the Score Test, and the number of significant covariates of 2^13^ = 8,192 CPH models are compared with each other.

## Figures and Tables

**Table 1.** Cross-validation results of Cox models, reporting mean and standard deviation of test metrics and variable p-values.

	1	2	3	4	5	6	7	8	9	10	Mean	SD
**GNS**	0.010	0.010	0.009	0.012	0.009	0.011	0.012	0.010	0.050	0.017	0.015	0.013
**HLE_s_**	0.013	0.032	0.007	0.023	0.029	0.032	0.011	0.045	0.024	0.011	0.023	0.012
**Stage**	0.007	0.004	0.011	0.028	0.008	0.047	0.009	0.034	0.007	0.039	0.019	0.016
**ETOH_no_**	0.065	0.023	0.117	0.094	0.146	0.200	0.036	0.046	0.081	0.031	0.084	0.057
**LRT**	2e-06	9e-07	2e-06	3e-05	6e-06	1e-04	1e-06	5e-06	2e-05	1e-05	3e-06	6e-06
**Score**	2e-06	3e-06	6e-06	4e-05	2e-05	3e-04	2e-06	6e-06	3e-05	2e-05	6e-06	1e-05
**CI_train_**	0.729	0.747	0.726	0.717	0.732	0.705	0.739	0.731	0.726	0.722	0.727	0.011
**CI_test_**	0.652	0.537	0.741	0.800	0.658	0.867	0.569	0.650	0.764	0.750	0.699	0.103

**Table 2.** Final model fitting on the sample data. 95% CI is calculated by exp (coef ± .96×se).

	*factor*	*coef.*	*H.ratio*	*se*	*95% CI*	*pvalue*
**Radiomics**	HLE_s_	0.259	1.29	0.103	[1.05, 1.58]	0.014

	GNS	−0.062	0.94	0.024	[0.90, 0.98]	0.009
	
**Clinical**	Stage	0.313	1.37	0.121	[1.08, 1.73]	0.009

	ETOH:2	−0.611	0.54	0.319	[0.29, 1.01]	0.055

	ETOH:3	−0.280	0.76	0.443	[0.32, 1.801	0.527

**Goodness of fit**	LRT Test	38.58				3e-07
	
	Wald Test	33.80				4e-04
	
	Score Test	36.94				6e-07

**Table 3.** Clinical characteristics summary

**Gender**	Male	79

	Female	70
	
**Race**	EA	133

	AA	16
	
**Smoking**	Yes	104

	No	34

	unknown	11

**Alcohol**	Yes	88

	No	43

	unknown	18

**AJCC Stage**	I	33

	II	21

	III	22

	IV	73
	
**Treatment Modality**	CRT	6

	CT	2

	Sx	87

	Sx + RT	27

	Sx + CRT	26

	Sx + CT	1
	
**Status**	Alive	83

	Dead	66

**Table 4.** Feature selection procedure

1.	Perform Pearson correlation analysis.
2.	Perform univariate score tests.
3.	Apply SparseL1 to constrain the degree of freedom.

## Data Availability

The datasets generated and/or analyzed during the current study are not publicly available due to institutional policy but are available from the corresponding author on reasonable request.
